# Farmed Areas Predict the Distribution of Amphibian Ponds in a Traditional Rural Landscape

**DOI:** 10.1371/journal.pone.0063649

**Published:** 2013-05-21

**Authors:** Tibor Hartel, Henrik von Wehrden

**Affiliations:** 1 Institute of Ecology, Faculty of Sustainability, Leuphana University Lüneburg, Lüneburg, Germany; 2 Sapientia University, Department of Environmental Sciences, Cluj-Napoca, Romania; 3 Ovidius University Constanta, Faculty of Natural and Agricultural Sciences, Constanţa, Romania; 4 Centre for Methods, Leuphana University Lüneburg, Lüneburg, Germany; 5 Research Institute of Wildlife Ecology, Vienna, Austria; Universität Zurich, Switzerland

## Abstract

**Background:**

Traditional rural landscapes of Eastern Europe are undergoing major changes due to agricultural intensification, land abandonment, change in agricultural practices and infrastructural development. Small man-made ponds are important yet vulnerable components of rural landscapes. Despite their important role for biodiversity, these ponds tend to be excluded from conservation strategies.

**Methodology/Findings:**

Our study was conducted in a traditional rural landscape in Eastern Europe. The aim of this study is twofold: (i) to model the distribution of four major man-made pond types and (ii) to present the importance of man-made ponds for the endangered Yellow Bellied Toad (*Bombina variegata*) and the Common Toad (*Bufo bufo*). Six environmental variables were used to model pond distribution: Corine landcover, the heterogeneity of the landcover, slope, road distance, distance to closest village and the human population density. Land cover heterogeneity was the most important driver for the distribution of fishponds. Areas used for agriculture with significant areas of natural vegetation were the most important predictors for the distribution of temporary ponds. In addition, areas covered by transitional woodland and scrub were important for the open cattle ponds. *Bombina variegata* was found predominantly in the temporary ponds (e.g. ponds created by cattle and buffalo, dirt road ponds and concrete ponds created for livestock drinking) and *Bufo bufo* in fishponds.

**Conclusions/Significance:**

Our Maxent models revealed that the highest probability of occurrence for amphibian ponds was in areas used as farmland. The traditional farming practices combined with a low level of infrastructure development produces a large number of amphibian ponds. The challenge is to harmonize economic development and the maintenance of high densities of ponds in these traditional rural landscapes.

## Introduction

Traditional rural landscapes are receiving increasing attention due to their high biodiversity [1]. In Eastern Europe in particular, traditional rural landscapes with high nature value farmland are being managed for agricultural production while still supporting comparatively high levels of biodiversity, including many species now endangered in other parts of Europe [2], [3]. It was recently highlighted that Eastern Europe should play its role in global food security [4], [5], and this trend will most likely trigger an increase in production intensity in the farmlands of the region [4].

Man-made ponds are typical components of agricultural landscapes. Many of these ponds are created and maintained for a variety of purposes, such as drinking sources for domestic animals, water sources for agricultural irrigation, fish production and places for recreational activities [6], [7]. Farmlands may be also rich in small temporary ponds, which are often created indirectly by various types of human and livestock activity. Despite their small size, a growing body of evidence suggests that permanent and temporary ponds significantly contribute to the biodiversity of entire landscapes [8]. Numerous protected or even endemic organisms are restricted to ponds within agricultural landscapes, including species of large branchiopods [9], amphibians [10], birds, plants and other organisms [11].

Due to their small size and dependency on human management, ponds and consequently the organisms inhabiting them are vulnerable to changes in land use practices. For example, ponds are threatened by land abandonment (i.e. through vegetation succession), land use intensification, the conversion of pastures into arable lands and the spread of urban areas [10], [12]. A rough estimation suggests that about half of European ponds disappeared in the 1900–1990 period due to these changes [12]. Despite the high levels of, often unique, biodiversity and their increasingly threatened status, ponds are still widely neglected in many countries both by conservation research and policy [8].

Amphibians depend on ponds and are therefore good model organisms to explore the human impact on ponds and associated wildlife. Amphibians use a large variety of farmland ponds, with some species preferring permanent fishponds while others are restricted to temporary ponds [10], [13]–[15]. Pond breeding amphibians have complex life cycles, which span over egg, larvae and post-metamorphic stages, and depend on small sized stagnant water bodies for reproduction [10], [16]. Due to their permeable skin, they are very sensitive to microclimatic, physical and chemical changes in their aquatic and terrestrial environment [17]. Migration and dispersal are key features of amphibian movements, with important consequences for population dynamics [16]. However, since their dispersal is rather slow, amphibians are extremely sensitive to habitat loss and fragmentation [18]. Consequently, amphibians are declining on a global scale, with European decline being mainly due to a loss of breeding and terrestrial habitats [19].

In this paper, we explore the factors governing the distribution of four types of man-made ponds in a traditional rural landscape in Transylvania (Romania). We use a number of variables related to land use, landcover, human population size and natural environment. Secondly, we show the ecological importance of these ponds by presenting the percent of occurrence of two amphibian species in these ponds: the Common Toad (*Bufo bufo*) and the internationally endangered Yellow Bellied Toad (*Bombina variegata*). Finally, based on our results we aim to highlight current challenges related to the maintenance of the pond networks in traditional rural landscapes.

## Materials and Methods

### Ethics Statement

This research was conducted within a broad research project targeting the Natura 2000 areas from Southern Transylvania, Romania (see Acknowledgements) lead by the World Wild Fund (WWF) in collaboration with other major local and regional institutions, with the permission of the administrators of the site (Progresul Silvic). No specific permissions were required for particular locations and activities presented in this paper. One of the species (the Yellow Bellied Toad – *Bombina variegata*) is protected according to national Law and Natura 2000 regulations. The research did not involve any harmful intervention to wildlife or the pond systems surveyed.

### Study Area

**Figure 1:**
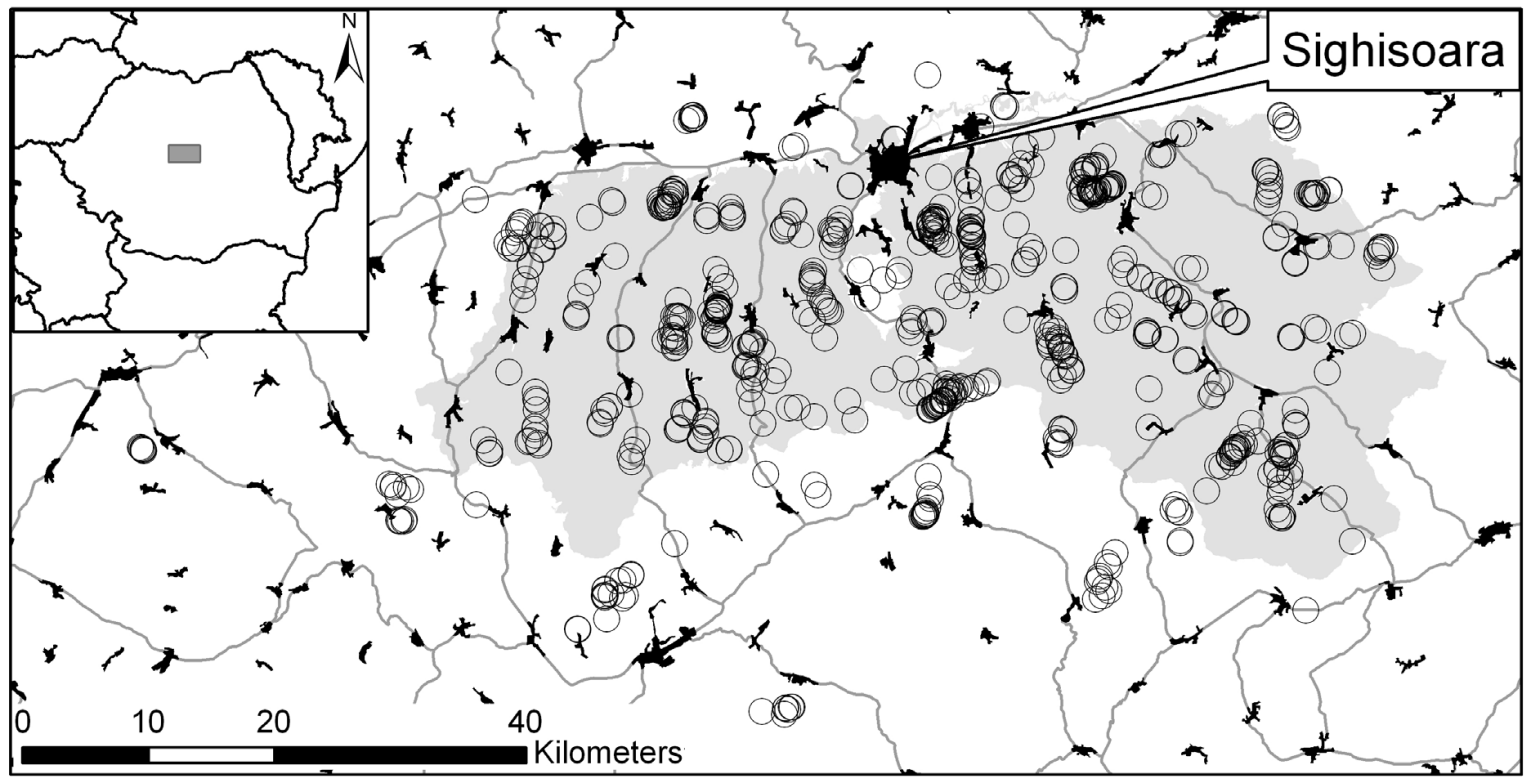
The location of the study region. Open circles represent the inventories ponds and the grey shade represents the Special Area for Conservation.

The study area is a hilly region of *ca* 3163 km^2^, located in Southern Transylvania, Romania, of which *ca* 860 km^2^ are under Natura 2000 regulations (Special Area of Conservation) (Fig. 1). The study area is dominated by traditional agricultural land use practices: the arable fields are still small and tilled mostly by horses and people similar to the management before mechanization of farming; artificial fertilizers and chemicals are rarely applied [3]. People use horses to extract wood from the forest and the infrastructural and urban development is overall low [3]. We refer to these agricultural practices in this paper from now on as being ‘traditional’. Currently ∼42% of the study area is covered by broad-leaved forest, ∼22% by meadows and pastures, ∼20% by arable fields. Built areas represent *ca* 3% of the region and other land use classes (e.g. wetlands, orchards etc.) add up to 100%. The number of permanent ponds under 1 ha area has increased recently in parts of the study area [20].

### Surveys

**Figure 2:**
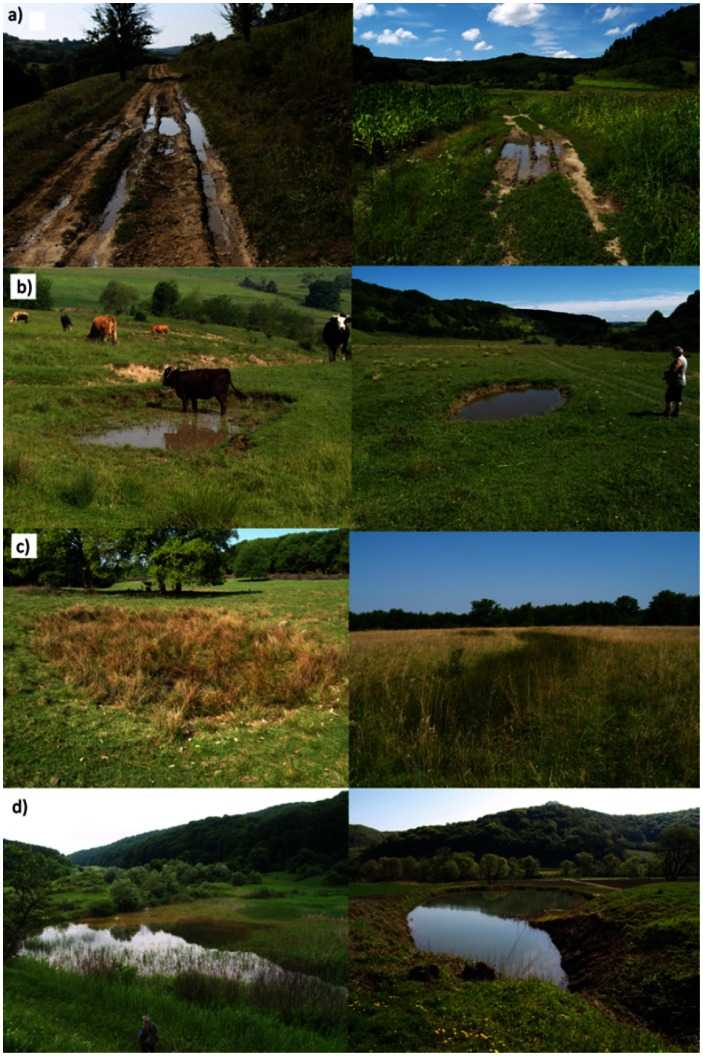
Sample pictures on the four pond type categories modelled in this paper: a) dirt road ponds, b) open cattle pond, c) overgrown ponds and d) fishponds. The first three categories are temporary ponds.

Pond surveys were carried out in 2011 (March–September) and 2012 (March–early July). The geographic coordinates and the altitude of each pond were measured with a handheld GPS device. Here we define as ‘ponds’ all small sized, permanent or temporary water bodies, which are non-flowing and are clearly distinguishable structures. We opportunistically but comprehensively sampled the whole surface area of the Natura 2000 site (see Fig. 1). Permanent ponds we located based on information from local people and satellite imagery (Google Earth). Some areas outside the Natura 2000 site were also surveyed (Fig. 1). In total 839 ponds were sampled. Each pond was classified into one of the following categories: (i) *dirt road ponds* (N = 377, Fig. 2) were the smallest temporary water bodies (*ca* 2–30 m^2^ surface area) created along unpaved roads mostly by horse carts and more rarely by heavy machinery such as tractors and machinery used for wood-extraction; dirt road ponds were the most abundant type of temporary ponds in this region. (ii) *Open cattle ponds* (N = 170, Fig. 2) were small sized (*ca* 4–200 m^2^) temporary ponds which were created by buffalo and cattle grazing and have low (i.e. <30%) vegetation coverage. Open cattle ponds indicate that there are a current cattle or buffalo grazing or were only recently discontinued. (iii) *Temporary ponds overgrown by vegetation* (‘overgrown’, N = 109, Fig. 2) were temporary ponds with similar size as the open cattle ponds (see above) but with more (i.e. > 60%) vegetation cover, especially with *Juncus* sp., *Carex* sp and *Lysimachia* sp. 95% of overgrown ponds were found within pastures where the grazing of cattle and buffalo had stopped or had severely decreased in its intensity. The hydroperiod of the temporary ponds is generally short. A five year study conducted in the centre of the study area [21] showed that the hydroperiod of temporary ponds ranged between 6 and 26 weeks. (iv) *Fishponds* (‘fish’, N = 37, Fig. 2) were small sized (i.e. *ca* 0.2–5 ha) permanent ponds created for recreation and livestock watering. Temporary ponds such as ditches (N = 51), livestock drinking troughs (N = 33) and various other small non-flowing water bodies with other origins (‘other’, (N = 62) were also inventoried. These were excluded from our modelling because they are not the result of clear land management and landuse strategies.

Amphibian surveys were started in the middle of March in both 2011 and 2012. Both species were very visible due to their abundance in the ponds, activity of their larvae and post metamorphic stages and the small size of the ponds. Amphibians were searched visually (this proved to be highly efficient in the small sized temporary ponds) and/or with dip netting. When finding at least one developmental stage in the water and/or close vicinity (i.e. up to *ca* two meter distance) we considered that the species was detected in the pond. Around 20% of the temporary ponds were surveyed two or more times in both years while the permanent ponds were surveyed once.

### Data Analysis

The environmental variables used to model pond distribution are presented in the Table 1. We used the maximum entropy algorithm “Maxent” to predict the distribution of different pond types within the study area. We used this modelling approach because its predictive power is comparable or even outperforms other available presence-only modelling algorithms [22]–[24]. Maxent randomly spreads background points to approximate the best fitting probability distribution in order to estimating habitat suitability [24]. These background points are then used for model evaluation using area under curve (AUC) statistics [22]; we used 100 runs and split the data 80%/20% for training and test datasets, respectively.

**Table 1:** The environmental variables used to model pond distribution.

Variable name	Description	Source
CORINE Land Cover classes (CLC)	44 landcover classes, out of which 20were occurring within our study area	European Environmental Agency (2011): (http://www.eea.europa.eu/publications/COR0-landcover).
Land cover heterogeneity	Landscape heterogeneity on a 1 ha scale	Calculated as the standard deviation within 1 ha circle using a nearest neighbor filter of the monochromatic channel of SPOT 5 data (©CNES 2007, Distribution Spot Image SA). Highest values for this variable occurred at the edge of two landuse types e.g. forest edge and pasture, or woody elements in an open area.
Slope	Slope in degrees	Calculated from Aster DEM data, downloaded from NASÀs Reverb (http://reverb.echo.nasa.gov/).
Road distance	Distance to the nearest road	Based on open source road layers (http://planet.openstreetmap.org).
Village distance	Distance to the nearest village	Based on open source village layers (http://planet.openstreetmap.org).
Human population density	Krigged based on commune polygons, where commune population was usedas a data basis	Based on population census data derived from national official databases.

We activated the “fade by clamping” option in Maxent to mitigate clamping issues to correct for extrapolation effects and spatially variable prediction results due to uneven data sampling [25], [26]. In order to spatially check for uneven prediction results we inspected the variability of the averaged model results; since overall prediction variability on average was low for all ponds we refrained from giving more details on this. In addition, we compared initial models based on random background points within the niche space of the presence points with background points matching the distribution of the presence points [26]. Since differences between both approaches were generally negligible across pond types (AUC difference <0.02), we used standard background points generated by 100 Maxent runs (10000 points) based on the niche space covered by the presence points. Results in the maps are predictions based on the area which is shown, thus partly projecting beyond the sampled area in order to show results for the whole region (see sample points in Fig. 1).

## Results

### Modelling Pond Distribution

**Figure 3:**
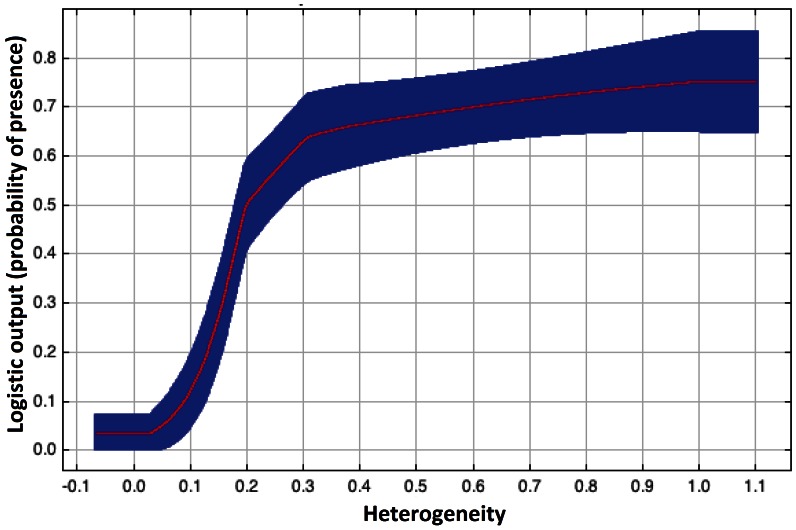
Distribution models for the four pond types addressed in this research. a) fishponds, b) open cattle ponds, c) dirt road ponds, d) overgrown ponds. Darker colours suggest higher probability of occurrence.

**Figure 4:**
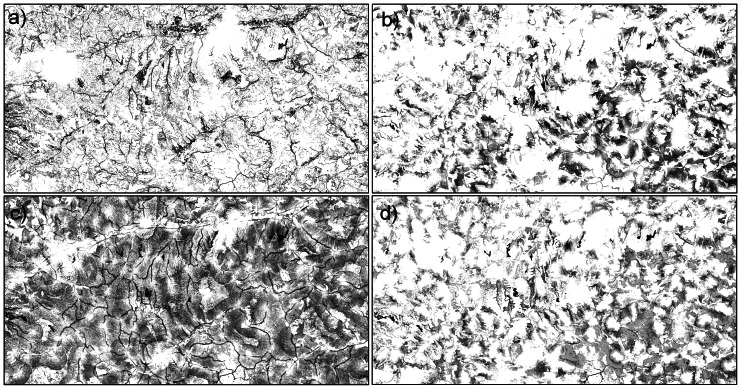
Response curve for the variable ‘heterogeneity’. The blue area corresponds to the standard deviation of 1.

Ponds were located at an average altitude of 526 m above sea level (minimum: 320 m; maximum: 774 m). The distribution model of ‘overgrown’ ponds had the highest accuracy (AUC = 0.82), while ‘fishponds’ and ‘open cattle’ pond showed intermediate values (AUC = 0.79 and 0.80). Distribution models for ‘dirt road ponds’ had the lowest AUC value (0.73) (see Table 2). Predictor contribution differed widely between different pond types (Table 2). Land use heterogeneity was the most important predictor for the permanent fishponds. The relationship between the variable heterogeneity and the probability of presence for fishponds is shown in Fig. 3. The Corine land cover variables were the most important predictor for the temporary ponds (Table 2). The landcover class ‘Land principally occupied by agriculture with significant areas of natural vegetation’ (CLC 243) was an important predictor for all pond types (Table 3). The ‘Transitional woodland and scrub’ (CLC 324), the landcover ‘Pasture’ (CLC 231) and ‘Natural grassland’ (CLC 321) were important predictors for the ‘fish ponds’ (Table 3). The landcover ‘Transitional woodland and scrub’ was important predictor for the ‘open cattle’ ponds (Table 3). The probability of finding ‘Dirt road ponds’ is relatively uniform across the region (excepting the human settlements) while the likelihood of finding other pond types is higher in certain areas of the region (Fig. 4).

**Table 2:** Percent contribution of different variables to the distribution model of four common pond types in Central Romania.

Pond type	CLC	Land coverheterogeneity	Slope	Roaddistance	Villagedistance	Human populationdensity	AverageAUC (SD)
‘Fish’	25	**49.2**	9.3	5.4	6.3	4.8	0.790 (0.10)
‘Dirt road pond’	**31.5**	14.2	7.2	21.7	12.8	12.6	0.737 (0.02)
‘Open cattle’	**41.4**	7.7	4.2	8.6	20.9	17.3	0.805 (0.03)
‘Overgrown’	**51.9**	9.1	6.1	6.5	17.6	8.9	0.826 (0.06)

**Table 3:** The importance of different landcover categories (as named in the Corine landcover database) for the distribution of the four pond types.

Pond type	Land principally occupied by agriculture withsignificant areas of natural vegetation	Pastures	Natural grasslands	Transitional woodlandand scrub^1^
‘Dirt road pond’	*			
‘Open cattle’	*			*
‘Fish pond’	*	*	*	*
‘Overgrown’	*			

* = important landcover classes with a presence probability>0.7.

^1^ = defined as wood-pastures in our study region.

The importance was assessed based on probability of occurrence of the given habitat classes in the Maxent results.

### Pond Use by Amphibians

**Figure 5:**
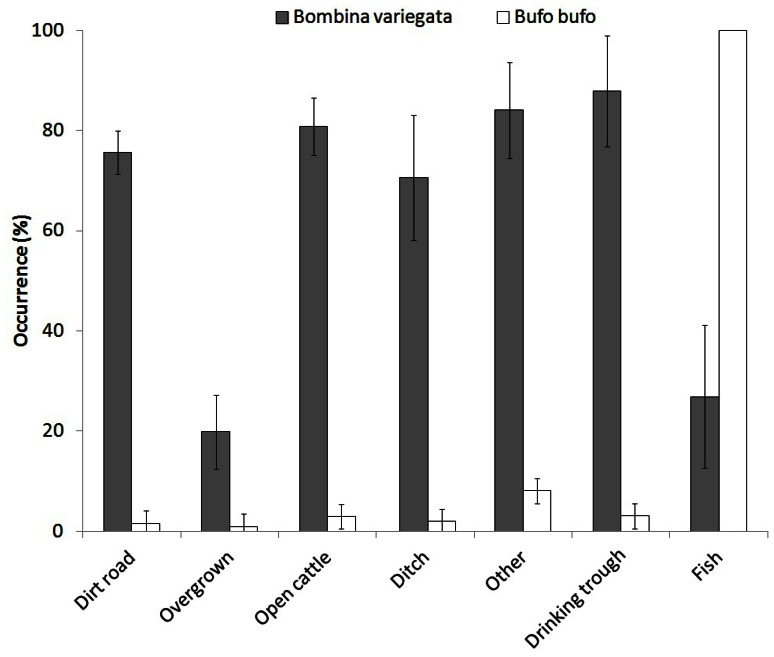
The occurrence (%) of *Bombina variegata* and *Bufo bufo* in different pond types based on the recorded presences. Errors are 95% confidence intervals. The only permanent ponds were the fishponds.

*Bombina variegata* was found in 603 ponds while *Bufo bufo* was found in 57, including all the ‘fishponds’. The occurrence of *B. variegata* was lowest in the ‘overgrown’ and ‘fishponds’ while *B. bufo* had low occurrence in the temporary ponds (Fig. 5).

## Discussion

Our analysis showed that landcover heterogeneity was the most important predictor for fishpond distribution. There are two potential explanations for this: (i) this may be an artefact of the permanent ponds themselves, which may contribute to the increased heterogeneity at local scale. We believe that this influence was overall small because the variable heterogeneity was influenced by the interface of more contrasting landuses (Table 1). (ii) An alternative explanation is that people deliberately select more heterogeneous landscapes for permanent pond creation. The number of small fishponds is sharply increasing in this region and interviews with fishpond owners suggest that they are important not only as a source of food and income, but also for recreation activities (*TH*, *unpublished data*).

Agricultural landcover representing farmed areas was an important driver for the distribution of all pond types modelled (Table 3). The forested areas were not important for the dirt road ponds although forest cover is high in the region (∼30% of land area–see the study area description) and many dirt road ponds were located in the forest. The most likely explanation is the relatively low heterogeneity of the forests, while agricultural landscapes show a more complex structure. People access their basic resources such hay, timber and crops mostly by horse carts and small tractors (these being overall very few in the villages) on dirt roads. This disturbance is the main cause of dirt road ponds. We found that land cover with transitional woodland and scrub were important predictors for the ‘open cattle ponds’. In our region these landscape elements are wood-pastures, which were traditionally grazed by cattle, buffalo and horses [27 and see Fig. 2]. Cattle prefer moist and more productive parts of the pasture [28], and moderate grazing with cattle contributes to the creation and maintenance of temporary wetlands and is often used in management strategies for wetland restoration (e.g. [29]–[31]); amphibians may benefit from this (e.g. [32], [33]).

We showed that even snapshot surveys can reveal high prevalence of two amphibian species in man-made ponds. The importance of man-made ponds for amphibians was reported from other regions of Europe, for example England [13], Sweden [34], Belgium [35], France [10], [14], Italy [36], Switzerland [37], Germany [38] and Romania [39]. *Bombina variegata* was frequently present in the surveyed temporary ponds in our study (Fig. 2). The high occurrence of *B. variegata* in traditionally managed rural landscapes might be explained both by its behaviour and the still high suitability of the whole region for temporary ponds as breeding habitats for this toad (e.g. Fig. 4b, c). *Bombina variegata* prefers temporary ponds and breeds multiple times during one season, usually in strong synchronization with rainfall, which fills the ponds [40]–[42]. This is possible because female *B. variegata* have continuous egg development [43]. *Bombina variegata* is capable of long-distance movements especially in the rainy periods of the year [44], [45]. This allows the colonization of newly formed (i.e. less than one year old) temporary ponds [45]. The eggs and larval periods are short [46] which is an advantage in exploiting ponds that only persist for a short duration.

*Bufo bufo* was found in all the fishponds surveyed while it occurs rarely in the temporary ponds. *Bufo bufo* prefers permanent ponds, which are typically surrounded by heterogeneous land cover in close proximity to forest [47]; the species is not sensitive to fish predation due to the unpalatable larvae [20], [48]. The reproductive success of *Bufo bufo* was however rather low in temporary ponds [21]. This species has good colonization ability [49] and the creation of additional fishponds may be beneficial for this species in the long run.

Traditionally managed farmlands are vulnerable to major changes in land use (e.g. intensification or abandonment, change in grazing livestock), infrastructural development and urbanization [50], [51]. Small temporary ponds, which depend on extensive landuse, may be among the first habitats that disappear if landuse is changed, intensified or the land is abandoned. Our study showed that a large number of small sized amphibian ponds (e.g. cattle ponds, dirt road ponds) are formed and maintained by traditional landuse practices and activities. Amphibian occurrence was high in these ponds. The currently applied land use practices in the rural communities of Southern Transylvania do not represent a free proactive choice for most of people; rather they are the result of poverty and lack of other options [3]. Local communities in this region have a strong desire to develop [52], making the conservation of small ponds and their associated biodiversity a challenging endeavour in this traditional rural region.
